# Up‐regulation of CHMP4B alleviates microglial necroptosis induced by traumatic brain injury

**DOI:** 10.1111/jcmm.15406

**Published:** 2020-06-25

**Authors:** Pengzhan Zhao, Chong Li, Binglin Chen, Guangchi Sun, Honglu Chao, Yiming Tu, Zhongyuan Bao, Liang Fan, Xiaoliu Du, Jing Ji

**Affiliations:** ^1^ Department of Neurosurgery the First Affiliated Hospital of Nanjing Medical University Nanjing China; ^2^ Department of Trauma Center the Third Affiliated Hospital of Nanchang University Nanjing Jiangxi China; ^3^ Department of Pathology the First Affiliated Hospital of Nanjing Medical University Nanjing China

**Keywords:** CHMP4B, FOXO1, microglia, necroptosis, traumatic brain injury

## Abstract

Microglial cells are key component of central nervous system (CNS) and mediate the immune response of the brain under physiological or pathological conditions. It tends to activate into a pro‐inflammatory M1 phenotype after traumatic brain injury (TBI) and promote secondary brain damage. Recently, necroptosis was found to promote microglial activation and neuroinflammation after TBI. However, the mechanism and specific interventions of microglial necroptosis after TBI remain poorly investigated. Here, we reported that overexpress the charged multivesicular body protein 4b (CHMP4B) which is a core member of the endosomal sorting required for transport complex III (ESCRT‐III) significantly decreased the level of necroptosis in microglia, improved neurological function recovery and protected against cell death after TBI. Further investigation showed that forkhead transcription factor O1 (FOXO1) was a crucial transcription factor that increased CHMP4B transcription by binding to the promoter region, thereby inhibiting necroptosis in microglia. Collectively, our findings demonstrated that CHMP4B relieved microglial necroptosis and neuroinflammation after TBI, and promote the recovery of nerve function. FOXO1 is an important factor in promoting CHMP4B expression. This study provides the novel viewpoint for TBI prevention and treatment.

## 1 INTRODUCTION

Traumatic brain injury (TBI) is common worldwide with high mortality and disability.^1^, ^2^ There are many causes of TBI, including fighting, traffic accidents and sports. The pathophysiological mechanisms of TBI include primary and secondary injuries that are difficult to treat. Secondary injury is characterized by biochemical and cellular changes, including inflammation, autophagy and apoptosis,^3^ which can lead to the mass death of neurons and thus affect the movement and sensation of patients. At present, clinical studies have shown that secondary brain injury is a feasible and important therapeutic target.^2^, ^4^, ^5^

Apoptosis, a type of cell death stimulated by proteases of the caspase family, has always been considered as a form of regulated cell death. In contrast, necrosis is thought to be an unregulated form of cell death.^6^, ^7^ However, with the progress of research, increasing evidence has shown that necrosis can also be regulated, similar to apoptosis, which is now called necroptosis.^7^ Necroptosis is initiated by activation of tumour necrosis factor alpha (TNFα) and/or Fas, which differentiates it from caspase‐dependent apoptosis.^8^ Necroptosis was recently observed in many nervous system pathologies, including brain trauma and cerebral ischaemia.^9^, ^10^ Long after the discovery of necroptosis, phosphorylated mixed lineage kinase domain‐like pseudokinase (p‐MLKL)‐labelled cell membranes were considered to indicate an irreversible cell death process that was not regulated by other factors.^11^, ^12^ However, current research has demonstrated that MLKL localizes to sites of broken membrane bubbles, where it recruits ESCRT‐III components, including CHMP4B, which in turn reduces cell membrane damage caused by p‐MLKL.^13^, ^14^

Some studies have proved that necroptotic cells induce a robust inflammatory response, in contrast to apoptotic cells, which are generally not associated with inflammation.^15^, ^16^, ^17^ Microglia are intrinsic immune effector cells in the central nervous system (CNS) that play important roles in both physiological and pathological processes. Studies have shown that microglia undergo necroptosis in the course of various nervous system injuries, such as intracerebral haemorrhage,^18^ spinal cord injury ^19^ and ischaemic stroke.^20^ Therefore, inhibiting microglial necroptosis may alleviate neuroinflammation and improve neuronal survival after traumatic brain injury.

Forkhead transcription factor O1 (FOXO1) is a member of the forkhead box‐containing transcription factor O family that participates in various cell physiological and pathological processes,^21^ including glycogenolysis and gluconeogenesis.^22^, ^23^ Except in glucose metabolism, FOXO1 has been shown to have a protective effect in organ damage. Some studies have reported that FOXO1 can alleviate cell damage, lower reactive oxygen species (ROS) levels and inhibit cell apoptosis in various pathological condition.^24^, ^25^ However, the role of FOXO1 in TBI and the underlying cellular and molecular mechanisms remain unclear.

In this study, we investigated the role of CHMP4B in brain injury. Interestingly, CHMP4B is involved in inhibiting necroptosis in microglia, in turn alleviating neuroinflammation and improving neuronal survival. Mechanistically, we found that FOXO1 regulates the expression of CHMP4B, in which knockdown of endogenous FOXO1 significantly reduces the transcription of CHMP4B. Thus, FOXO1 participates in the anti‐necroptotic effect of CHMP4B.

## 2 MATERIALS AND METHODS

### 2.1 Patients

The Department of Neurosurgery at the First Affiliated Hospital of Nanjing Medical University provided human brain tissue (Table 1), which was approved by the Institutional Review Board. Brain tissues resected from patients with severe TBI were snap‐frozen and stored in liquid nitrogen until assay. Brain tissues resected from epilepsy patients were used as control. The Ethics Committee of Nanjing Medical University approved the use of human brain tissue, and all procedures were conducted in accordance with approved guidelines. The participant's explicit permission was obtained, and the patient provided informed consent.

**TABLE 1:** Clinical information of human brain specimens

No. of patient	Gender	Age	Diagnosis	Time to injury	Site
Ctrl 1	Male	52	Epilepsy	‐	Right temporal lobe
Ctrl 2	Male	21	Epilepsy	‐	Right temporal lobe
Ctrl 3	Male	68	Epilepsy	‐	Left temporal lobe
TBI 1	Male	53	TBI	4 h	Left frontal lobe
TBI 2	Male	62	TBI	6 h	Left frontal lobe
TBI 3	Male	33	TBI	5 h	Left temporal lobe

Note

Abbreviations: Ctrl, control; TBI, traumatic brain injury.

### 2.2 Animals

Experimental Animal Central of Nanjing Medical University provided adult male C57BL/6J mice (25 ± 2 g). We arranged all mice five per cage under humid environment and at regulated temperature (22 ± 2°C) in a light and dark cycle of 12:12 hours. Mice received standard laboratory animal food and water. The Institutional Animal Care and Use Committee of Nanjing Medical University authorized all experiments conducted in accordance with the ‘Principles of Laboratory Animal Care’ (National Institutes of Health Publication No. 85‐23, revised 1996).

### 2.3 Experimental TBI model

As described previously, the TBI groups underwent surgery to produce a controlled cortical impingement model.^26^ Mice underwent anaesthesia with 4% isoflurane in 70% N_2_O and 30% O_2_. Anaesthesia was maintained with 2%‐3% isoflurane. Then, in the left parietotemporal cortex (the relative coordinates centre of craniotomy to bregma: 1.5 mm posterior and 2.5 mm lateral), we performed a 5 mm craniotomy with an electric drill. For the sham groups, only the dura mater was exposed. In the TBI groups, the exposed dura mater was struck by a 3‐mm metal impounder at 6 m/s velocity with 1 mm set depth and 100‐ms impacting period. We closed the scalp by suturing, and then mice were caged. The mice were then decapitated at various times, and only the cerebral cortex around the lesion was collected and stored in 4% paraformaldehyde or at −80°C for the following study.

### 2.4 Primary cortical neuronal culture

The SPF grade one‐day‐old C57BL/6J mice were used for this study. The mice were decollated immediately after sterilizing the skin with 75% alcohol, and the intact brain was immersed in pre‐cooled DMEM/F12 medium. The meninges and blood vessels were removed from a thin layer of cerebral cortex and placed in a fresh pre‐cooled DMEM/F12 medium, and then minced into small pieces with ophthalmic scissors. The tissue was then digested with 0.25% trypsin and DNase at 37°C for 20 minutes (with shaking every 5 minutes). The digestion was stopped by the addition of horse serum and filtered through a 70 μm cell strainer. The filtrate underwent centrifugation at 1000 rpm for 5 minutes, and the supernatant was discarded. Then, we resuspended the cell pellet in plating medium DMEM/F12 medium containing 10% horse serum + 100 U/mL penicillin + 1% glutamine. After mixing, the cell suspension was filtered again, and after adjusting the cell concentration, the cells were plated onto 6‐ or 12‐well cell culture dishes coated with L‐polylysine at a density of 1 × 10^6 ^cells/mL and placed in a 37°C, 5% CO_2_ cell incubator. After 4 hours, the medium was replaced with neuronal medium (96% neurobasal medium + 2% B27 + 100 U/mL penicillin + 1% glutamine) after the neurons were adherent. The medium was replaced with fresh medium every 2‐3 days, and using an inverted microscope, we monitored the cells’ growth status. The cells were used for experiments after approximately 7 days in culture.

### 2.5 Adeno‐associated virus (AAV) intracerebroventricular injection

According to the process described in the previous study.^27^ We anesthetized the mice with 4% chloral hydrate. Besides, AAV underwent injection to the left hemisphere (anteroposterior, 0.23 mm; lateral, 1.0 mm; depth, 2.2 mm; from bregma) in an intracerebroventricular manner (i.c.v.) with the use of stereotaxic approaches a week prior to TBI. Applying a stepper‐motorized microsyringe (Hamilton, Bonaduz, Switzerland) at 1 μL/min, the injecting processes were conducted. In addition, we adopted amoxicillin for preventing infection caused by bacterium. The control mice were administrated the identical dose, whereas the adenoviruses only carried luciferase. Our CHMP4B‐overexpressed AAV (GenePharma) encompassed the NM_029362.3:157‐831 Mus musculus CHMP4B, 5′‐ATGTCGGTGTTCGGGAAGCTGTTCGGGGCTGGAGGGGGTAAGGCGGGCAAGGGCGGCCCGACCCCCCAGGAGGCCATCCAGCGGCTTCGGGACACGGAGGAGATGTTAAGCAAGAAGCAGGAGTTCCTGGAGAAGAAAATCGAACAGGAGCTGACGGCTGCCAAGAAGCACGGCACCAAAAATAAACGCGCCGCCCTGCAGGCTCTGAAGCGCAAGAAGAGGTATGAGAAGCAGCTGGCACAAATTGATGGCACCCTGTCAACCATCGAGTTCCAGCGGGAGGCCCTAGAGAACGCCAACACCAACACGGAGGTGCTCAAGAACATGGGCTATGCCGCCAAGGCCATGAAGGCTGCCCACGACAACATGGACATTGATAAGGTGGATGAGTTAATGCAGGACATTGCTGACCAGCAAGAACTTGCAGAGGAGATTTCCACAGCTATCTCCAAACCTGTGGGCTTTGGAGAAGAGTTCGACGAGGATGAGCTCATGGCAGAGTTGGAGGAACTTGAACAAGAGGAGTTGGACAAGAATTTGTTGGAGATCAGTGGGCCCGAAACAGTCCCTCTACCAAATGTCCCCTCCGTAGCCCTACCATCCAAACCCGCCAAGAAGAAGGAAGAGGAAGATGACGACATGAAGGAATTGGAGAACTGGGCCGGATCCATGTAA‐3′.

### 2.6 BV2 cell culture, transfection and in *vitro* injury model

The BV2 cell line was purchased from the Chinese Academy of Sciences Cell Bank and was cultured in Dulbecco's modified Eagle's medium (DMEM). All of the medium were mixed with penicillin, streptomycin and 10% foetal bovine serum (FBS) (Gibco). The cultivation environment was maintained at 37°C and 5% CO_2_. Generally, the BV2 cells were used for the following experiments when the growth density reaches 70%‐80%. The BV2 cells were transfected with the indicated plasmids using Lipofectamine 3000 (Invitrogen). After 24 hours, we treated BV2 cells with 100 μm glutamate (Glu) for inducing cellular injury for 24 hours according to the study protocol.

### 2.7 Western blotting

Cytosolic, cytomembrane and overall extracts of protein were requested following the previous description.^28^, ^29^ Proteins at the site of trauma and on the contralateral side were harvested at different time and extracted using radioimmunoprecipitation assay (RIPA) buffer on ice (Sigma) and then centrifuged for 15 minutes 1000 *g* 4°C. The proteins underwent separation using 12% SDS‐PAGE gel and placed to polyvinylidene fluoride (PVDF) membranes (Millipore). Membranes underwent incubation with 5% (w/v) non‐fat dried milk for 2 hours at ambient temperatures and then incubated using primary antibodies throughout the night at 4°C. The membranes underwent incubation in the antibodies below: GAPDH (1:2000, #5174; Cell Signaling Technology), RIP3 (1 µg/mL, ab62344; Abcam), p‐MLKL (1:500, ab208910, Abcam), CHMP4B (1 µg/mL, ab105767; Abcam) and FOXO1 (1:1000, ab39670; Abcam). Using an enhanced chemiluminescence detection system (GE Healthcare), we ascertained bound antibodies. Applying ImageJ software (National Institutes of Health), we studied the achieved bands’ optical density.

### 2.8 Immunohistochemistry

Brain sections were incubated overnight with primary antibodies at 4°C. Subsequently, the sections underwent incubation in a minor antibody (Abcam) at 37°C for 30 minutes, rinsing processed using phosphate‐buffered saline and then incubation using an avidin‐biotin‐peroxidase complex at 37°C for 30 minutes. Primary antibodies were RIP3 (5 µg/mL, ab62344; Abcam), p‐MLKL (1:100, JM92‐37; Novus Biologicals) and CHMP4B (1:1000, ab135154; Abcam).

### 2.9 Immunofluorescence staining

The frozen brain sections were permeabilized for 20 minutes with 0.2% Triton X‐100 (Sigma‐Aldrich, St Louis, MO; USA, X100); then, they underwent blocking processed with 5% normal goat serum (Millipore; S26‐LITER) and incubation throughout the night using major antibodies and then using minor antibodies for 2 hours at ambient temperatures. Nuclei underwent staining processed using DAPI. Major antibodies included RIP3 (5 µg/mL, ab62344; Abcam), p‐MLKL (1:100, ab208910, Abcam), CHMP4B (5 µg/mL, ab105767; Abcam) and FOXO1 (1‐5 µg/mL, ab39670; Abcam). Cell processing was like sections, except that they underwent initial fixing processed for 20 minutes with pre‐cooled paraformaldehyde (4%, w/v). Using an Olympus FluoView confocal microscope with appropriate emission filters (Olympus), we observed immunoreactivity.

### 2.10 TUNEL assay

Apoptosis in 8‐μm frozen brain sections was examined by TUNEL staining according to the manufacturer's directions (ISCDD, Boehringer Mannheim). Brain sections were incubated with 50 μL TUNEL reaction mixture for 1 hour at ambient temperature in a room without light. After the incubating process, the slides were rinsed three times with PBS (pH 7.4) and stained with DAPI to detect nuclei with blue fluorescence. Similarly, NeuN was stained with Anti‐NeuN antibody (1:200, ab190195; Abcam) and ascertained by green fluorescence. By TUNEL assay, apoptotic cells displayed red fluorescence. Separated TUNEL‐positive cells underwent counting process in 1 mm^2^ field in the trauma area of the five mice sampled randomly from each group. An observer blinded to the research designing process counted TUNEL‐positive cells’ number in each of the 20 consecutive fields of view in five sections of each mouse, and the average number of TUNEL‐positive cells/NeuN in the field of view was calculated for each mouse.

### 2.11 Magnetic resonance imaging (MRI)

On the 3 weeks after TBI, three animals were taken in a random manner from every group for MRI examination. Mice underwent anaesthesis with halothane (3%‐4% induction, 1.5%‐2% maintenance) in oxygen (0.4 L/min) and nitrogen (0.6 L/min). A mouse having undergone anaesthesis was arranged prone on the fixation system and ascertained with a small animal MRI system (Bruker BioSpec7T/20 USR; Bruker AXS GmbH). We performed the sequence protocol with the following parameters: T2‐weighted; 256 × 256 matrix; slice thickness, 1 mm; intersection gap, 1 mm; echo time/repetition time: 27/3000 milliseconds; rapid acquisition with relaxation enhancement factor, 16; and flip angle, 90°. We generated T2‐weighted images in sagittal and axial planes with ParaVision (version 6.0.1, Bruker BioSpec; Bruker AXS GmbH).

### 2.12 Brain water content

After sacrifice, the mice brain underwent immediate removal and weighing process. Subsequently, it underwent drying process at 70°C for 72 hours to achieve the dry weight. The brain water content was obtained based on water content (%) = [(wet weight − dry weight)/wet weight] × 100%.^30^

### 2.13 Morris water maze

Each experimental animal was taken to a behavioural room kept at 25°C (housing area during training period). After a two‐day adaptation period, the training lasted seven days. The water was made opaque by the addition of milk. In brief, the mice were given 90 seconds to find the platform, and if the mouse could not reach the platform within 90 seconds, the researcher placed it on the platform for 15 seconds for spatial learning and orientation memory training. The first 5 days were used for training, and four trials were conducted, and the mice were released from the four different quadrants for each trial. On day 6, the time to the platform from each quadrant was recorded. If the platform was not found, a time of 90 seconds was recorded. On day 7, the platform was removed, and the time spent in the target quadrant and the number of times the mouse crossed the target platform location was recorded as indexes of learning and memory. In the study, a researcher with no knowledge of each mouse's experimental groups performed the behavioural tests.

### 2.14 Rotarod test

The mice were allowed to walk for three days on a rotating rod at 10 revolutions every min (RPM). On the 4th day, we assessed the ability of each groups of mice to walk on rotarod. Rotarod was accelerated from 4 rpm to 40 RPM for 5 minutes, and we recorded the time for each mouse to fall off the rod to be the retention time. Every mouse was tested three times, and the data were statistically analysed.

### 2.15 ELLSA assay

TNF‐α, IL‐6 and IL‐1β expressions in culture supernatants were measured by enzyme‐linked immunosorbent assay (ELISA; R&D Systems Inc) according to the manufacturer's instruction.

### 2.16 Cellular fractionation

According to the manufacturer's instructions, nuclear/plasma separation kits (Norgen Biotek Corp.) were used for nuclear/plasma separation. The efficiency of cellular fractionation was tested by cytoplasmic and nuclear markers TH and UBF, respectively.

### 2.17 RNA extraction and qRT‐PCR

Total RNA was extracted from brain and BV2 cell line using TRIZOL reagent (Invitrogen). cDNA was produced using the PrimeScript‐RT Reagent Kit (Takara). qRT‐PCR was performed using the SYBR Green PCR kit (Takara) on an ABI 7500 real‐time PCR system (Applied Biosystems). The results were normalized to β‐actin mRNA expression and analysed using the 2^−ΔΔCt^ method. The specific primers were synthesized by (Ribobio): mouse CHMP4B primers, forward 5′‐ATGTCGGTGTTCGGGAAGC‐3′ and reverse 5′‐CGGCGCGTTTATTTTTGGTG‐3′; mouse FOXO1 primers, forward 5′‐ CCCAGGCCGGAGTTTAACC‐3′ and reverse 5′‐ GTTGCTCATAAAGTCGGTGCT‐3′; mouse β‐actin primers, forward 5′‐ACTGCCGCATCCTCTTCCT‐3′ and reverse 5′‐TCAACGTCACACTTCATGATGGA‐3′

### 2.18 Dual‐luciferase gene reporter assay

In order to analyse the transcriptional activity of CHMP4B, we generated a 1000 base pair (bp) genome fragment corresponding to the CHMP4B promoter region by PCR and cloned it into the pGL3 luciferase reporter vector (Promega). BV2 cells were cotransfected with the reporter vector and beta‐galactosidase cDNA by Lipofectamine 3000. The cells were lysed in a lysis buffer provided by the luciferase assay kit (Promega) after 48 hours transfection, and the luciferase activity was detected by a MicroLumat Plus LB96V luminometer (Berthold Technologies). The luciferase activity was normalized to beta‐galactosidase activity in relative light units (RLUs).^31^

### 2.19 Chromatin immunoprecipitation (ChIP)

Purification of sonicated nuclear lysates and immunoprecipitation were performed using the Simple Chip Enzymatic Chromatin IP kit (Cell Signaling Technology) according to manufacturer's instructions.

### 2.20 Flow cytometric analysis

Nerve cells were cultured in 6‐well plates and subjected to different treatments. Cells were trypsinized with 0.25% trypsin (without EDTA), centrifuged at 160 *g* for 5 minutes and resuspended in 300 μL of binding buffer. Subsequently, the cell suspension was stained with 3 μL Annexin V‐APC and 3 μL 7‐AAD (KGA1023, KeyGEN biotech). After 20 minutes incubation, cells were analysed using a flow cytometer.

### 2.21 Cell viability assay

Using the Cell Counting Kit‐8 (CCK‐8, CK04, Dojindo) assay, we ascertained cell viability. Major neurons underwent seeding process in 96‐well plates at a density of 1.5 × 10^4^ cells/mL. We transfected cells with various lentivirus (Flag‐CHMP4B; shS100A11) for 3 days, and then, the cells were treated with 100 μmol/L glutamate for 24 hours for inducing cellular injury. We introduced the CCK‐8 diluted to the working concentration to 96‐well plates and incubated kept them for 4 hours at 37°C. We ascertained the absorbance at 450 nm using a Thermo Multiskan FC microplate photometer.

### 2.22 Lactate dehydrogenase assay

Cell death was measured by lactate dehydrogenase (LDH) release kit in line with the directions of the manufacturer (C0016, Beyotime Biotech).

### 2.23 Statistical analysis

Results of images and data are reported to be means ± SEM from more than three separated experimental processes. Grey levels were detected with ImageJ. Variance underwent two‐way ANOVA analysis for determining the diversification between treating processes, and non‐parametric *t* test was adopted for comparing the diversifications of the two groups. *P*< .05 was considered statistically significant

## 3 RESULTS

### 3.1 Necroptosis after clinical and experimental TBI

At present, it is generally accepted that receptor‐interacting protein kinase‐3 (RIPK3) and phosphorylated mixed lineage kinase domain‐like pseudokinase (p‐MLKL) constitute the core of the necroptosis machinery termed as the necrosome.^26^, ^27^, ^28^ We hypothesized that the severity of CNS damage after TBI is related to the levels of RIP3 and p‐MLKL.

**FIGURE 1:**
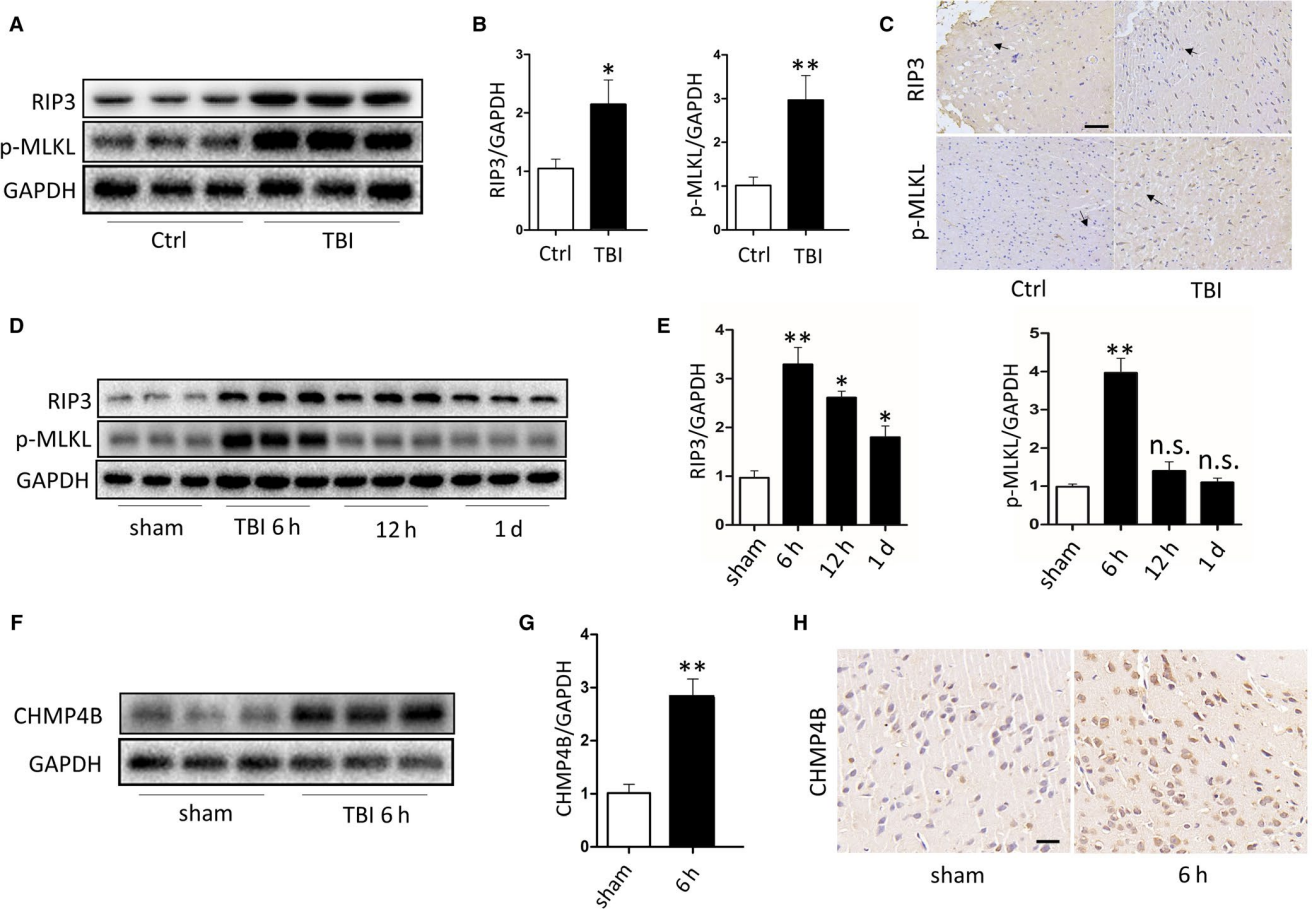
There is necroptosis after traumatic brain injury and its level changes with time. A, Protein levels of RIP3 and p‐MLKL obtained from epileptic patients and severe trauma patients. GAPDH was used as the loading control. B, Bar graphs show the results of analysis (by band density analysis) of RIP3 and p‐MLKL (trauma patients = 3, epileptic patients = 3; data are presented as the means ± SEM). C, Immunohistochemistry of RIP3 and p‐MLKL in TBI and Ctrl. (Scale bar = 100 μm.) D, Protein levels of RIP3 and p‐MLKL obtained from sham mice and TBI mice at 6 h, 12 h and 1 d. GAPDH was used as the loading control. E, Bar graphs show the results of analysis (by band density analysis) of RIP3 and p‐MLKL (TBI = 5, sham = 5; data are presented as the means ± SEM). F, Protein levels of CHMP4B obtained from sham mice and TBI mice at 6 h. GAPDH was used as the loading control. G, Bar graphs show the results of analysis (by band density analysis) of CHMP4B (TBI = 5, sham = 5; data are presented as the means ± SEM). H, Immunohistochemistry of CHMP4B in sham and TBI at 6 h. (Scale bar = 50 μm.) *P < .05; **P < .01

We used Western blot analysis to compare the levels of RIP3 and p‐MLKL in severe trauma patients with intractable intracranial pressure and control patients. Obviously, increased levels of RIP3 and p‐MLKL were observed in trauma patients compared with controls (Figure 1A,B). Furthermore, immunohistochemistry showed that RIP3 and p‐MLKL levels were significantly higher in trauma patients than controls (Figure 1C).

In TBI, necroptosis has been found to be involved in the process of CNS tissue damage. However, the temporal peak of necroptosis may vary because of differences in the mouse models used. Here, we found that the level of necroptosis varied over time in our experimental TBI model. Western blot analysis showed that RIP3 and p‐MLKL protein contents in the traumatized cortex were markedly increased at 6 hours after TBI compared with the sham groups. However, 24 hours after TBI, RIP3 and p‐MLKL protein levels decreased markedly compared with 6 hours (Figure 1D,E). These data show that necroptosis peaks at 6 hours in the traumatized cortical area after TBI.

In view of the above results, the CHMP4B protein level was detected at 6 hours in the traumatized cortex. Western blotting and immunohistochemistry revealed that CHMP4B protein levels were almost threefold higher than in sham mice (Figure 1F‐H).

### 3.2 CHMP4B exerts protective effects after TBI

To explore the role of CHMP4B in TBI, we injected mice with adeno‐associated virus (AAV), either AAV‐CHMP4B (carrying CHMP4B) or AAV‐vector at 1 week before TBI. Infection of the virus was demonstrated by optical in vivo imaging assay, qRT‐PCR analysis and Western blotting (Figure S1A‐D). Only transfected mice were used in the subsequent experiments.

**FIGURE 2:**
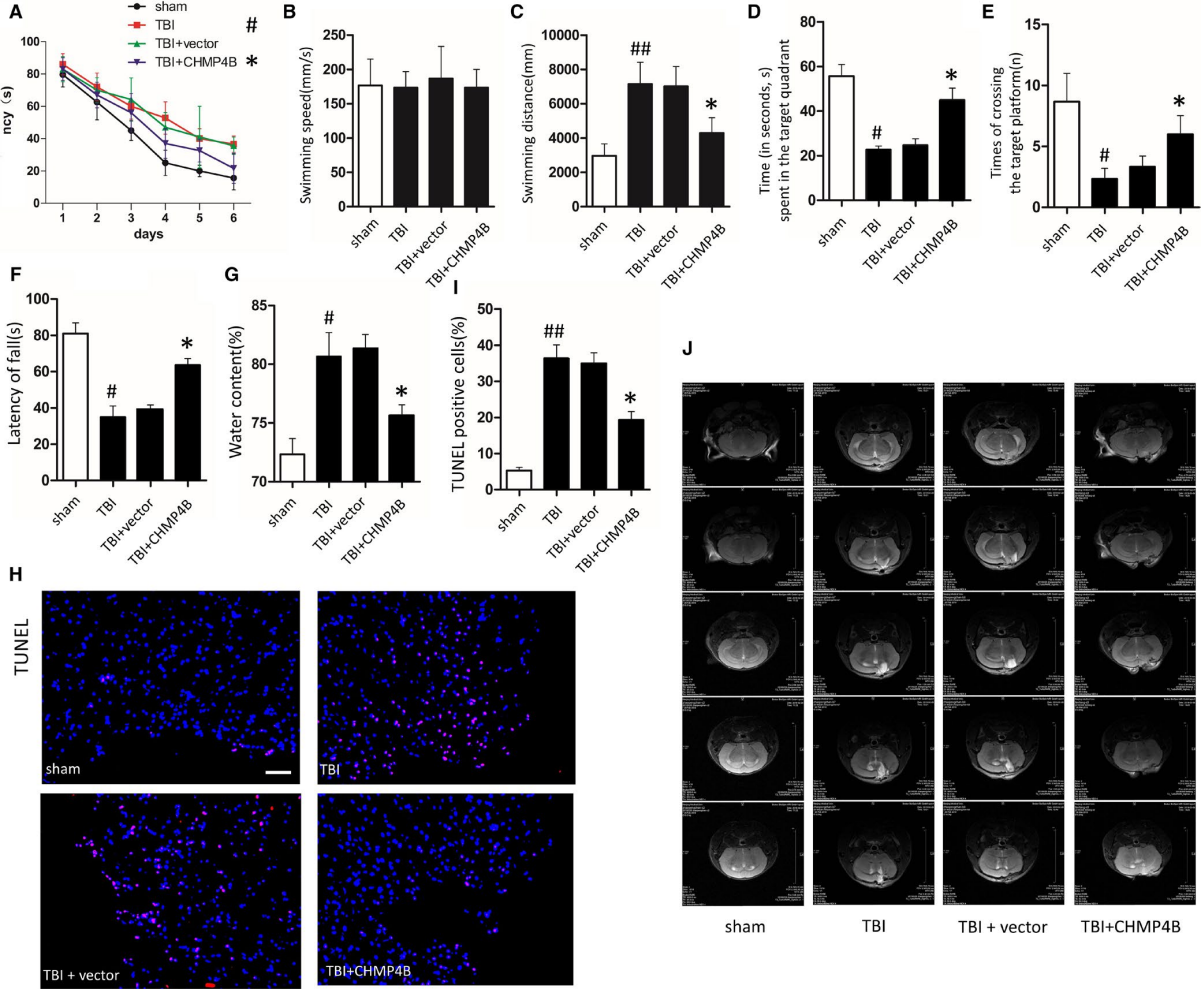
CHMP4B improves memory and motor ability and reduces the number of cell death and the size of oedema area after TBI. There are four treatment groups take part in the experiments: sham, TBI, TBI + vector and TBI + CHMP4B (#P: TBI groups compared with sham groups; *P: TBI + CHMP4B groups compared with the TBI + vector groups). A, Mean latency to the platform of each group over 6 d (n = 10 mice; data are presented as the means ± SEM). B and C, Mean swimming speed (B) and distance (C) of each group on the day 6 (n = 10 mice; data are presented as the means ± SEM). D and E, Time (s) spent in the target quadrant (D) and the number of times the mouse crossed the target platform location (E) during the probe trials on day 7 (n = 10 mice; data are presented as the means ± SEM). F, The time mice can stay on the rotarod (n = 10 mice; data are presented as the means ± SEM). G, Brain water content after TBI (n = 3 mice; data are presented as the means ± SEM). H, Representative images of TUNEL staining after TBI in each group (Scale bar = 100 μm). I, Statistical analysis of the positive cell shown in G (n = 5 mice; data are presented as the means ± SEM). J, Mouse brain tissue MRI T2W1 images in each group. *P < .05; #*P* < .05, ##*P* < .01

To examine memory retention, Morris water maze tests were performed to assess cognitive deficits at 3 weeks after injury. In the first 2 days, all the groups treated with TBI performed poorly compared with the sham groups. Over the next 4 days, the TBI + AAV‐CHMP4B group performed significantly better. Contrast, the TBI and TBI + AAV‐vector groups continued to perform poorly and the improvement is not obvious than TBI + AAV‐CHMP4B group (Figure 2A). On the last day, compared with TBI and TBI + AAV‐vector groups, the swimming distance of TBI + AAV‐CHMP4B group was significantly ameliorated (Figure 2B,C). Next, we removed the platform and observed the performance on day 7. The TBI + AAV‐CHMP4B group crossed the platform more often and spent more time in the target quadrant than the TBI groups and TBI + AAV‐vector groups (Figure 2D,E). The rotarod test, which tests the motor ability, revealed that TBI + AAV‐CHMP4B groups remained longer on the rotarod than the TBI and TBI + AAV‐vector groups (Figure 2F).

Brain water content was significantly decreased in the TBI + AAV‐CHMP4B group compared with the TBI and TBI + AAV‐vector groups (Figure 2G).

Furthermore, terminal deoxynucleotidyl transferase dUTP nick end labelling (TUNEL)‐positive cells were examined in the traumatized area after TBI. The numbers of TUNEL‐positive cells in TBI + AAV‐CHMP4B group were markedly decreased in comparison with the TBI and TBI + AAV‐vector groups (Figure 2H,I).

Moreover, the cortical lesion volume and oedema area were significantly decreased in the TBI + AAV‐CHMP4B group compared with the TBI and TBI + AAV‐vector groups after 3 weeks (Figure 2J).

**FIGURE 3:**
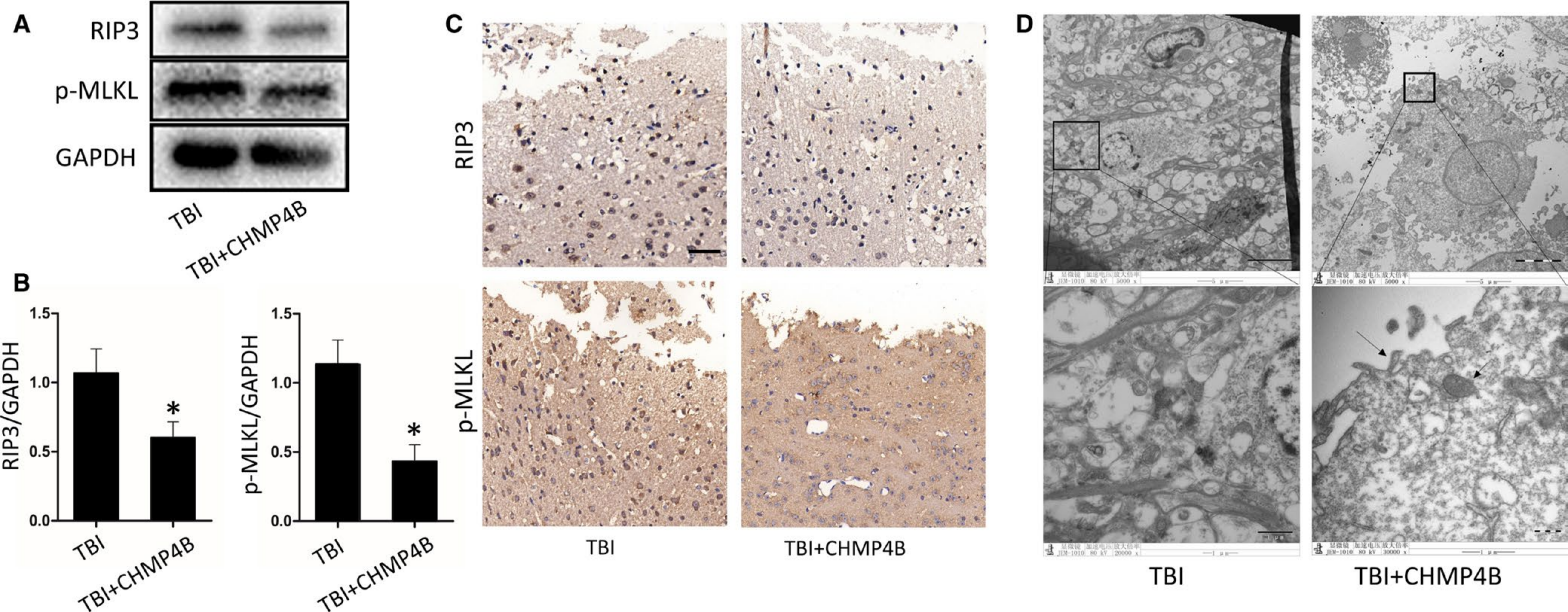
CHMP4B alleviates the level of necroptosis after TBI. A, Protein levels of RIP3 and p‐MLKL obtained from TBI group and TBI + CHMP4B group. GAPDH was used as the loading control. B, Bar graphs show the results of analysis (by band density analysis) of RIP3 and p‐MLKL (n = 5 mice; data are presented as the means ± SEM). C, Immunohistochemistry of RIP3 and p‐MLKL in TBI group and TBI + CHMP4B group. (Scale bar = 50 μm.) D, Transmission electron microscopy (TEM) images of tissues. Translucent cytoplasm mitochondrial swelling and destruction of membrane integrity were observed in TBI neurons. (Scale bar = 5 or 1 μm.) *P < .05

We continue to study the role of CHMP4B on necroptosis, and the results of Western blotting and immunohistochemistry reflected that TBI + AAV‐CHMP4B group had lower level of necroptosis compared with the TBI group (Figure 3A‐C). Finally, we used transmission electron microscopy (TEM) to observe cells’ morphology. TBI + AAV‐CHMP4B group showed a denser cytoplasm, more intact cell membrane and less mitochondria swelling than TBI group (Figure 3D).

These results demonstrate that up‐regulation of CHMP4B enhances recovery of motor and memory functions, reduces cell death and alleviates the necroptosis after TBI in mice.

### 3.3 CHMP4B plays a protective role by alleviating the necroptosis in microglia

**FIGURE 4:**
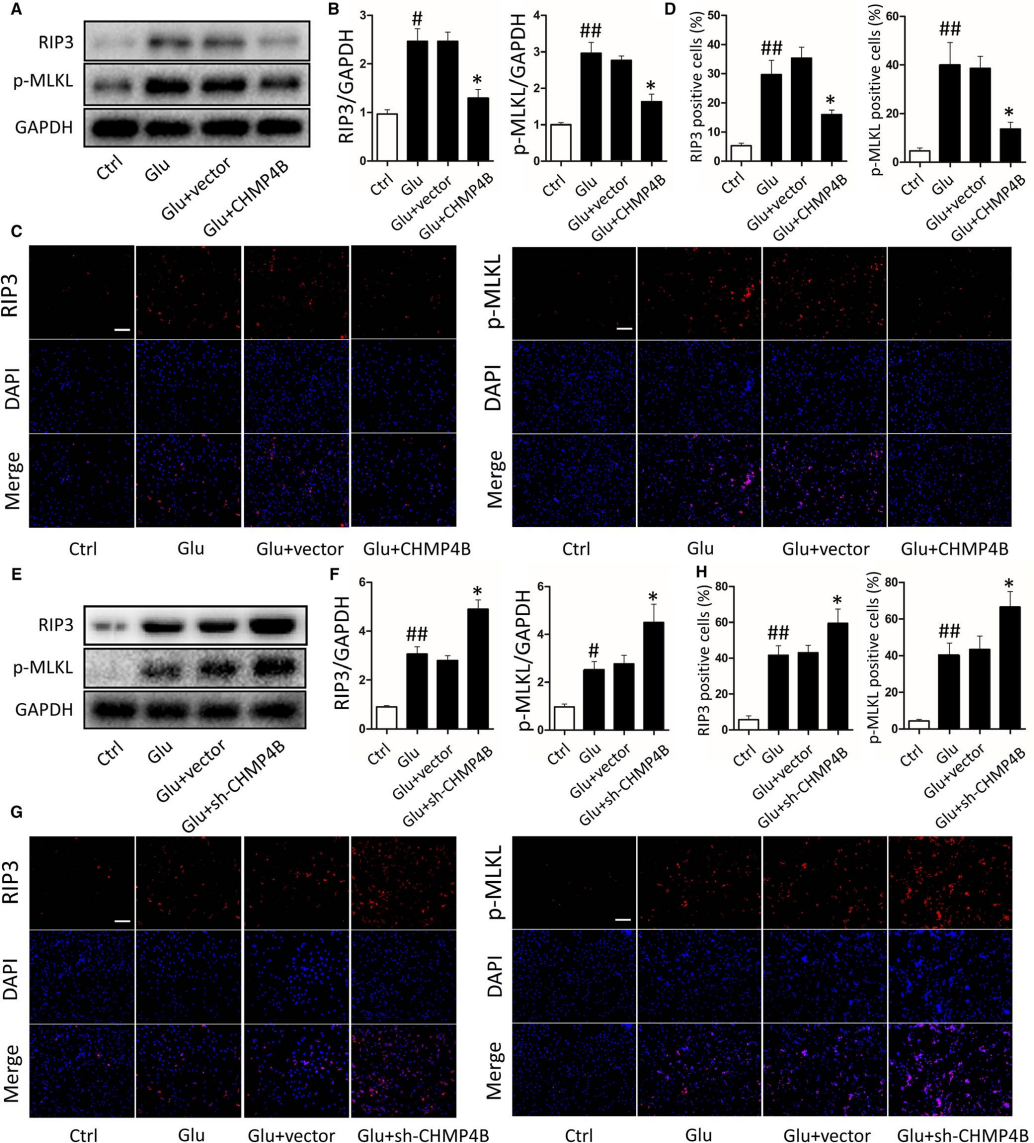
Effects of CHMP4B on necroptosis in BV2 cells. There are four treatment groups take part in the experiments: control, Glu, Glu + vector, Glu + CHMP4B (#*P*: Glu groups compared with Ctrl groups; *P: Glu + CHMP4B groups compared with the Glu + vector groups). A, Protein levels of RIP3 and p‐MLKL. GAPDH was used as the loading control. B, Bar graphs show the results of analysis (by band density analysis) of RIP3 and p‐MLKL (n = 3; data are presented as the means ± SEM). C, Immunofluorescence of RIP3 and p‐MLKL (scale bar = 150 μm). D, Statistical analysis of the positive cells shown in C (n = 3; data are presented as the means ± SEM). There are four treatment groups take part in the experiments: control, Glu, Glu + vector, Glu + sh‐CHMP4B (#*P*: Glu groups compared with Ctrl groups; *P: Glu + sh‐CHMP4B groups compared with the Glu + vector groups). E, Protein levels of RIP3 and p‐MLKL. GAPDH was used as the loading control. F, Bar graphs show the results of analysis (by band density analysis) of RIP3 and p‐MLKL (n = 3; data are presented as the means ± SEM). G, Immunofluorescence of RIP3 and p‐MLKL (scale bar = 150 μm). H, Statistical analysis of the positive cells shown in G (n = 3; data are presented as the means ± SEM). *P < .05; #*P* < .05; ##*P* < .01

Previous studies have shown that activated microglia increase inflammation in the CNS and reduce the survival of neurons after injury.^32^ To clarify the molecular mechanisms underlying the neuroprotective effect of CHMP4B, we performed experiments on BV2 cells. To simulate a cellular model of trauma, we treated BV2 cells with glutamate (Glu) to induce cell injury.^33^ Meanwhile, we transfected the plasmid which overexpressed CHMP4B or empty vector into microglia (Figure S2A‐C). Glu treatment increased the expression of RIP3 and p‐MLKL as well as the number of activated BV2. When CHMP4B was overexpressed in these cells, the levels of the necroptosis marker proteins were reduced, as assessed by Western blotting (Figure 4A,B). The results of immunofluorescence also reflected the same phenomenon, namely CHMP4B overexpression reduced the number of necroptotic cells in comparison with the Glu and Glu + vector groups (Figure 4C,D).

Next, three independent plasmids encoding short hairpin RNAs targeting CHMP4B were constructed to silence CHMP4B expression in microglia. qRT‐PCR and Western blot analyses indicated that sh‐CHMP4B #1 had the highest interference efficiency and was therefore chosen for the following experiments (Figure S2D‐F). Western blotting showed theGlu + sh‐CHMP4B group had higher levels of the necroptosis marker proteins than the Glu and Glu + vector groups. (Figure 4E,F) Then, we performed the results by immunofluorescence. Again, the Glu + sh‐CHMP4B group had a higher level of the necroptosis markers than the Glu and Glu + vector groups (Figure 4G,H).

These results suggest that CHMP4B decreases necroptosis in injured BV2 cells.

### 3.4 CHMP4B suppresses activated microglia‐mediated injury to neurons

Given the involvement of CHMP4B in the inhibition of necroptosis, we next explored its effect on the survival of neurons. To this end, we added microglial‐conditioned medium (MCM) to primary cortical neurons.^34^ MCM from Glu‐treatment microglia, but not resting MCM (Glu group), noticeably affected the growth of neurons. In comparison, MCM from activated microglia pretreated with vector overexpressing CHMP4B (Glu + CHMP4B group) did not have a harmful effect.

**FIGURE 5:**
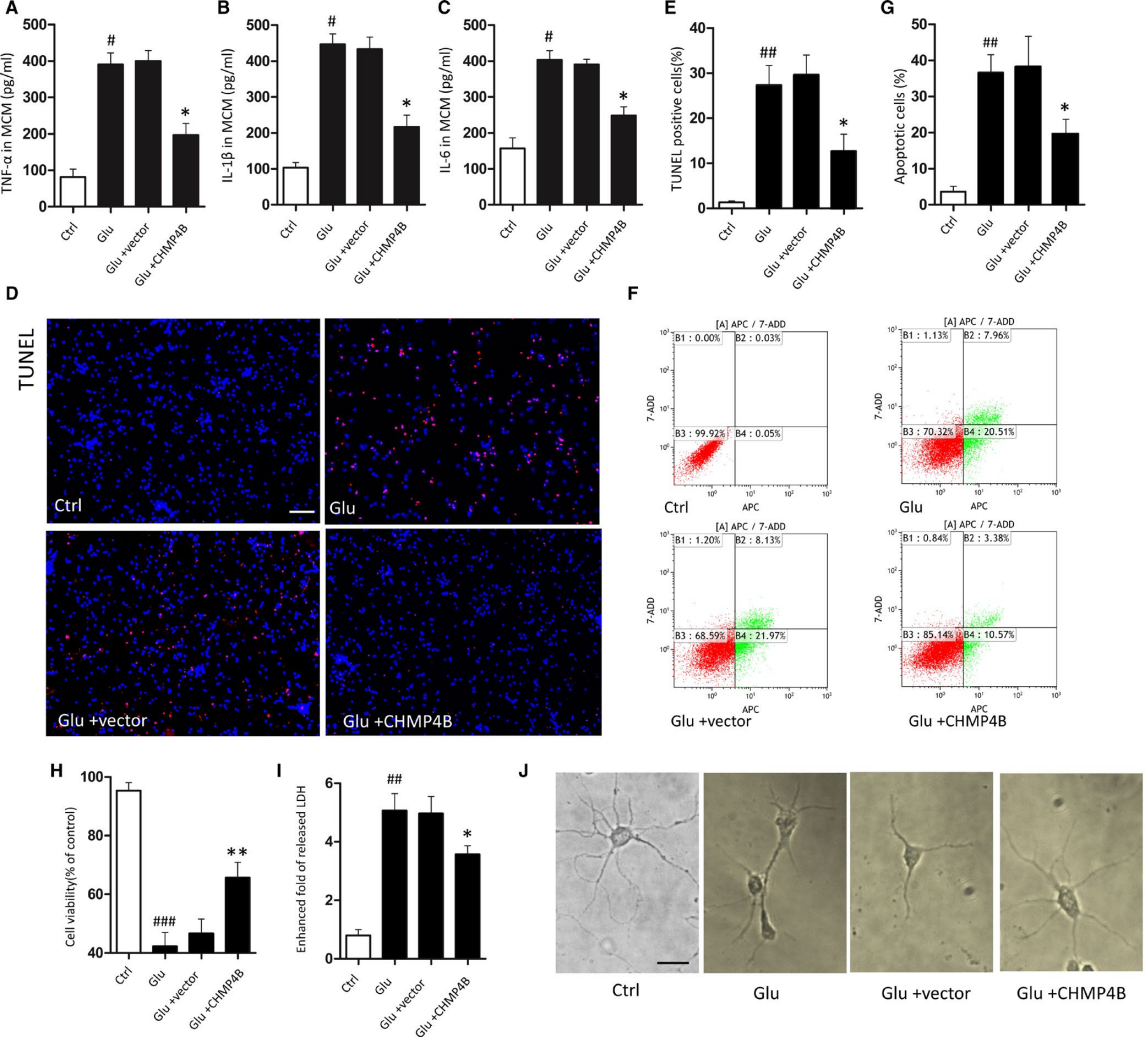
CHMP4B inhibited the neuronal toxicity of activated microglia. There are four treatment groups take part in the experiments: control, Glu, Glu + vector, Glu + CHMP4B (#*P*: Glu groups compared with Ctrl groups; *P: Glu + CHMP4B groups compared with the Glu + vector groups). A‐C, MCM levels of TNF‐α (A), IL‐1β (B) and IL‐6 (C). D, Representative images of TUNEL staining after injury in each group (Scale bar = 150 μm). E, Statistical analysis of the positive cells shown in A (n = 3; data are presented as the means ± SEM). F, Representative flow cytometry images of neuron stained with Annexin V‐APC /7‐AAD. G, The apoptosis activation was represented by ratio of APC + (n = 3; data are presented as the means ± SEM). H, CCK‐8 assay tested primary neurons viability (n = 5; data are presented as the means ± SEM). I, Lactate dehydrogenase release assay (n = 3; data are presented as the means ± SEM). J, Representative images of neurons growth one week after different treatments (scale bar = 20 μm). *P < .05; **P < .01; #*P* < .05, ##*P* < .01, ###*P* < .001

We first measured concentrations of TNF‐α, IL‐1β and IL‐6 in MCM. The ELLSA results showed that these inflammatory factors were significant higher in Glu group than in the Glu + CHMP4B group (Figure 5A‐C).

Next, we examined TUNEL‐positive cells after stimulation. A marked reduction in TUNEL‐positive neurons was observed in the Glu + CHMP4B group compare with the Glu group (Figure 5D,E). Flow cytometry corroborated these results (Figure 5F,G). Moreover, MCM from microglia overexpressing CHMP4B improved the viability of neurons after stimulation compared with the Glu groups (Figure 5H). Furthermore the Glu group showed the more fold of released LDH, whereas theGlu + CHMP4B group showed a comparatively lower release of LDH (Figure 5I).

Thereafter, we examined the growth of neurons. After stimulation, neurons displayed axonal shortening and aggregation. When cultured with MCM from microglia treated with Glu and overexpressing CHMP4B, neuronal appearance remained normal (Figure 5J).

Based on these results, CHMP4B can reduce a variety of harmful secretions from activated microglia.

### 3.5 FOXO1 regulates the mRNA and protein levels of CHMP4B after TBI

**FIGURE 6:**
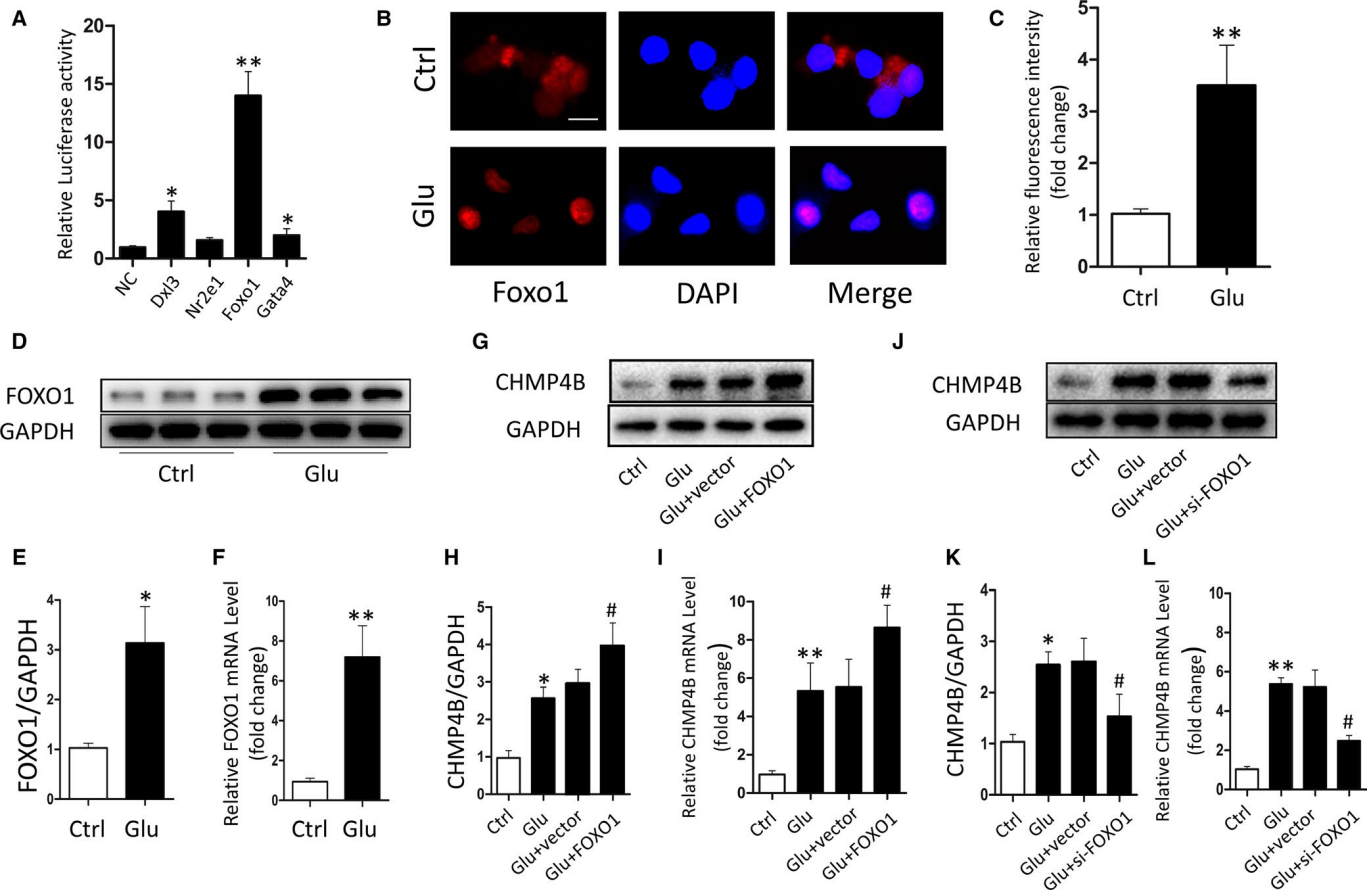
FOXO1 regulates CHMP4B transcription and expression. A, The relative luciferase activity in BV2 cells after co‐transfected plasmids containing transcription factors and CHMP4B. B and C, Expression of nuclear FOXO1 was detected by laser scanning confocal microscopy (scale bar = 20 μm). D, Protein level of FOXO1. GAPDH was used as the loading control. E, Bar graphs show the results of analysis (by band density analysis) of FOXO1 (n = 3; data are presented as the means ± SEM). F, qRT‐PCR analysis of FOXO1 expression in Ctrl groups and Glu groups (n = 3; data are presented as the means ± SEM). G‐L. BV2 cells were transfected with FOXO1 plasmid or siRNA for 2 d, and then added the Glu. The protein and mRNA levels of CHMP4B were determined by Western blot and qRT‐PCR (n = 3; data are presented as the means ± SEM; *P: Glu groups compared with Ctrl groups; #*P*: Glu + FOXO1 groups compared with the Glu + vector groups). *P < .05; **P < .01; #*P* < .05

Many studies have confirmed that changes in protein expression can be attributed to upstream transcriptional inducers when tissues or cells are stimulated.^35^ According to the JASPAR database prediction, Dxl3, Nr2e1, Foxo1 and Gata4 had the highest prediction scores. Then, we sought to validated this database prediction by luciferase assay and found that FOXO1 had the strongest luciferase inducing activity among them (Figure 6A). Thus, FOXO1 was selected for the subsequent studies.

The immunofluorescence assay showed a higher fluorescence intensity of FOXO1 in nuclei after stimulation (Figure 6B,C). And using qPCR and Western blotting, we found that the level of FOXO1 expression increased in the nuclei of BV2 cells after treatment with pro‐apoptotic stimuli (Figure 6D‐F). Next, we increased or decreased FOXO1 expression in BV2 cells by transfection with pcDNA‐FOXO1 or si‐FOXO1, respectively. qRT‐PCR analysis and Western blotting were used to verify the transfection efficiency (Figure S3). Next, we examined the effect of FOXO1 on the changes of CHMP4B expression. CHMP4B expression was reduced or increased according to FOXO1 levels. Furthermore, FOXO1 overexpression increased the mRNA and protein levels of CHMP4B after Glu stimulation compared with Glu stimuli alone (Figure 6G‐L). These results suggest that FOXO1 promotes the transcription of CHMP4B, thereby increasing CHMP4B protein levels after proapoptotic stimulation.

Moreover, overexpression of FOXO1 significantly decreased the level of necroptosis after treatment of BV2 cells with Glu. In contrast, knockdown of FOXO1 increased the levels of necroptosis after Glu stimulation (Figure S4).

### 3.6 FOXO1 regulates CHMP4B at the transcription level by binding to the promoter region

**FIGURE 7:**
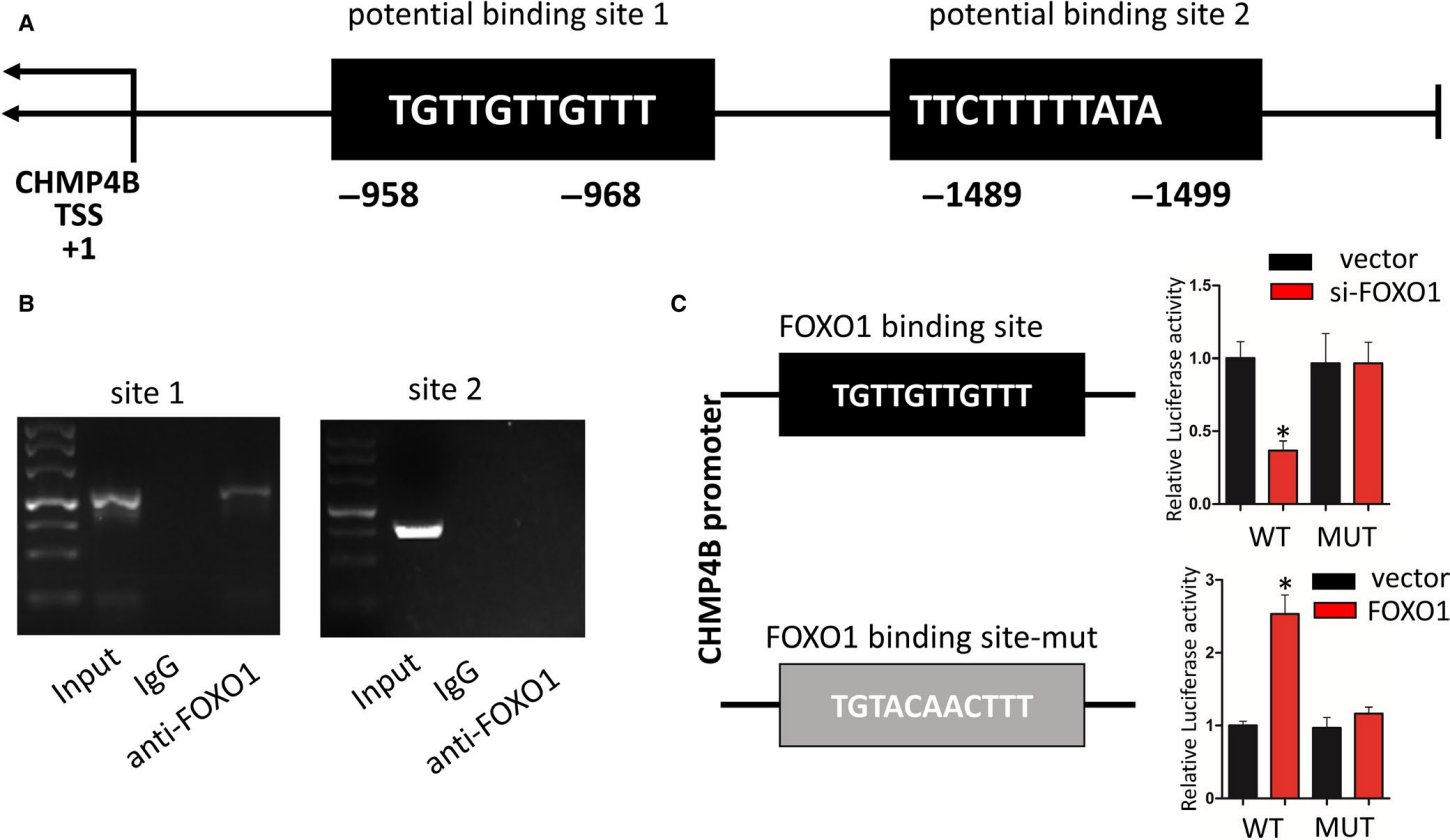
FOXO1 binds the CHMP4B promoter region. A, Schematic diagram revealing the mouse CHMP4B promoter region and FOXO1 potential binding site. B, ChIP assay results determined the relative enrichment of FOXO1 on promoter region of CHMP4B. C, Schematic view represented WT and mutant Rip3 promoter. And luciferase reporter assay was performed in BV2 cells cotransfected FOXO1 or si‐FOXO1 with WT and mutant Rip3 promoter (n = 3; data are presented as the means ± SEM). *P < .05

To further explore the regulatory interaction between FOXO1 and CHMP4B, we explored the promoter region of CHMP4B with the JASPER database, and three potential binding sites with the highest prediction scores were selected (Figure 7A). The ChIP assay revealed that FOXO1 bounds to CHMP4B only at Site 1, the −958 to −968 bp region (Figure 7B). Finally, we verified the binding site of FOXO1 on the CHMP4B promoter by generating mutations. As expected, luciferase assay showed that the transcriptional activity of the CHMP4B promoter is significantly increased, when the CHMP4B promoter binding by FOXO1 (Figure 7C).

These results indicate that FOXO1 directly enhances CHMP4B transcription by binding to a specific region on the CHMP4B promoter.

## 4 DISCUSSION

Traumatic brain injury is common in neurosurgery, and not only results in high mortality and disability, but is also considered to contribute to chronic diseases such as Alzheimer's disease, chronic traumatic encephalopathy and Parkinson's disease.^29^, ^30^ However, there is still no treatment that has been proven to be effective in improving prognosis of TBI and reducing complications.^29^ In the pathophysiological process of TBI, microglia are activated first, in turn promoting neuroinflammation and secondary brain injury.^34^ Therefore, a better understanding of the microglial activation mechanism is urgently needed for better treatment of TBI. Based on accumulating evidence, necroptosis occurs in the acute phase after TBI and can cause massive loss of neurons, which is a major neuropathological event. Traditionally, necroptosis is triggered by the TNF signalling pathway, which has been reported to be involved in neurobehavioural and histological outcomes after TBI.^36^, ^37^ When necroptosis is triggered, receptor‐interacting protein (RIP) 1 and its cognate kinase RIP3 are recruited to form supramolecular complexes, including the TNFR‐associated death domain (TRADD), FAS‐associated protein with death domain (FADD) and caspase‐8, known as the necrosome, which is thought to function as a platform for necroptosis.^38^ And RHIM‐dependent autophosphorylation of RIPK3 leads to recruitment and phosphorylation of MLKL,^39^, ^40^ which contributes to the conformational change of the pseudokinase domain, eventually leading to cell rupture by changing the permeability of the plasma membrane.^41^, ^42^ Long after the discovery of necroptosis, MLKL‐labelled cell membranes had been considered to indicate an irreversible cell death process that was not regulated by other factors.^13^ However, current research has shown that MLKL localizes to sites of ruptured membrane bubbles, where it recruits ESCRT‐III components, including CHMP4B, which reduces cell membrane damage by reversing the adverse effect of MLKL.^14^ These studies prompted us to search for potential targets for neuroprotection against necroptosis after TBI. The ESCRT family, especially CHMP4B, reduces damage caused by p‐MLKL, thereby preventing cell death.^13^ However, the role of CHMP4B has not been explored in brain trauma. Thus, we hypothesized that CHMP4B may be an important factor inhibiting necroptosis during TBI.

To validate this hypothesis, in this study, we explored changes in the expression and activity of key necroptotic proteins in brain tissue from human. We found that the expression of RIP3 and p‐MLKL increased after TBI compared with epileptic tissue. Then, we produced a TBI mouse model to further investigate these change. Western blotting showed that the RIP3 and p‐MLKL content peaked after 6 hours, which is during a relatively early phase. Thereafter, RIP3 and p‐MLKL levels gradually decreased. Coincidentally, at 6 hours after TBI, the expression of CHMP4B also increased compared with normal tissues, thus indicating that there may be some connection between CHMP4B and necroptosis in TBI. Furthermore, we examined the effects of various treatments and the role of CHMP4B in TBI. The results indicated that CHMP4B overexpression decreased RIP3 and p‐MLKL‐positive cells. More importantly, CHMP4B overexpression in *vivo* improved motor and cognitive functions in mice after TBI, and at the same time, decreased cell death. Thus, we conclude that necroptosis occurs in the early phase of TBI, and CHMP4B can inhibit necroptosis and promote CNS recovery.

In subsequent in vitro experiments, we treated BV2 cells with Glu to induce cell injury. Our results confirmed that overexpression of CHMP4B inhibited necroptosis after microglial injury and reduced the pro‐inflammatory effect of microglia, thereby alleviating neuronal damage. As previously reported, the expression of proteins is regulated by upstream transcription factors.^35^ By a database prediction, we obtained a number of potential candidate regulators. Among these, FOXO1, a classical transcription factor, had the strongest luciferase inducting ability. We found that FOXO1 increased CHMP4B transcription by binding to the promoter region (located at −1499 to −1489 bp) in microglia. Stable knockdown of FOXO1 can reduce the expression of CHMP4B, thereby increasing the level of necroptosis after microglia damage.

Previous studies have shown that the endosomal sorting complexes required for transport III (ESCRT‐III) are involved in many physiological and pathological processes including the multivesicular body (MVB) pathway, cytokinesis, viral budding, plasma membrane repair and apoptosis.^43^, ^44^, ^45^ Although it has been confirmed that CHMP4B affects neuronal growth, differentiation and apoptosis, its role in TBI had remained unknown. Here, we found that in TBI, CHMP4B inhibits necroptosis and reduces cell death, and its protective effect is greater than its adverse effect.

There are several limitations to this study. First, we used a TBI model employing juvenile mice, whereas the clinical samples were obtained from adult patients. Differences in age and species may affect the generalizability of our findings. Second, our microglial injury model was induced by Glu. Although it is a widely used model, the physiological and pathological processes may differ from those of traumatic injury. Third, for the clinical samples, epileptic patients were used as controls and were therefore not true healthy controls. However, because it is unethical to remove brain tissue from a normal healthy control, they represent the best available source of control samples. In addition, the clinical TBI tissues were mainly from the frontal lobe, whereas those in the control groups were all from the temporal lobe. Furthermore, the mean age of the control patients was lower than that of the TBI patients. Nevertheless, they represent the best control simples available for this study.

## 5 CONCLUSION

In summary, we found that CHMP4B effectively reduces necroptosis after TBI. And FOXO1 functions as a transcriptional activator that binds CHMP4B at its promoter region to induce expression. Inhibition of TBI‐induced necroptosis improves motor and memory functional recovery in mice. Our findings are important to better understand the mechanisms underlying secondary injury after TBI and may lead to the discovery of new necroptotic inhibitors as potential new therapies for TBI and other acute CNS injuries.

## CONFLICT OF INTEREST

The authors declare that they have no competing interests.

## AUTHOR CONTRIBUTION

Jing Ji and Pengzhan Zhao designed this research. Pengzhan Zhao,Binglin Chen, and Guangchi Sun performed and analyzed the experiments. Honglu Chao and Yiming Tu conceived the experiments. Zhongyuan Bao, Liang Fan, Xiaoliu Du and Chong Li assisted with data analysis. Pengzhan Zhao, Binglin Chen, and Chong Li wrote the manuscript. All authors reviewed the manuscript.

## Supporting information

FigS1‐S4

## Data Availability

All data generated or analysed in the current study are included either in this article or in the additional files.
