# Supplementary material for: Up‐regulation of CHMP4B alleviates microglial necroptosis induced by traumatic brain injury
Source: J Cell Mol Med. 2020 Jun 25;24(15):8466–79. doi: 10.1111/jcmm.15406 (PMC7412706; doi:10.1111/jcmm.15406)
Supplement: Supplementary file 1 — FigS1‐S4 [file JCMM-24-8466-s001.docx]

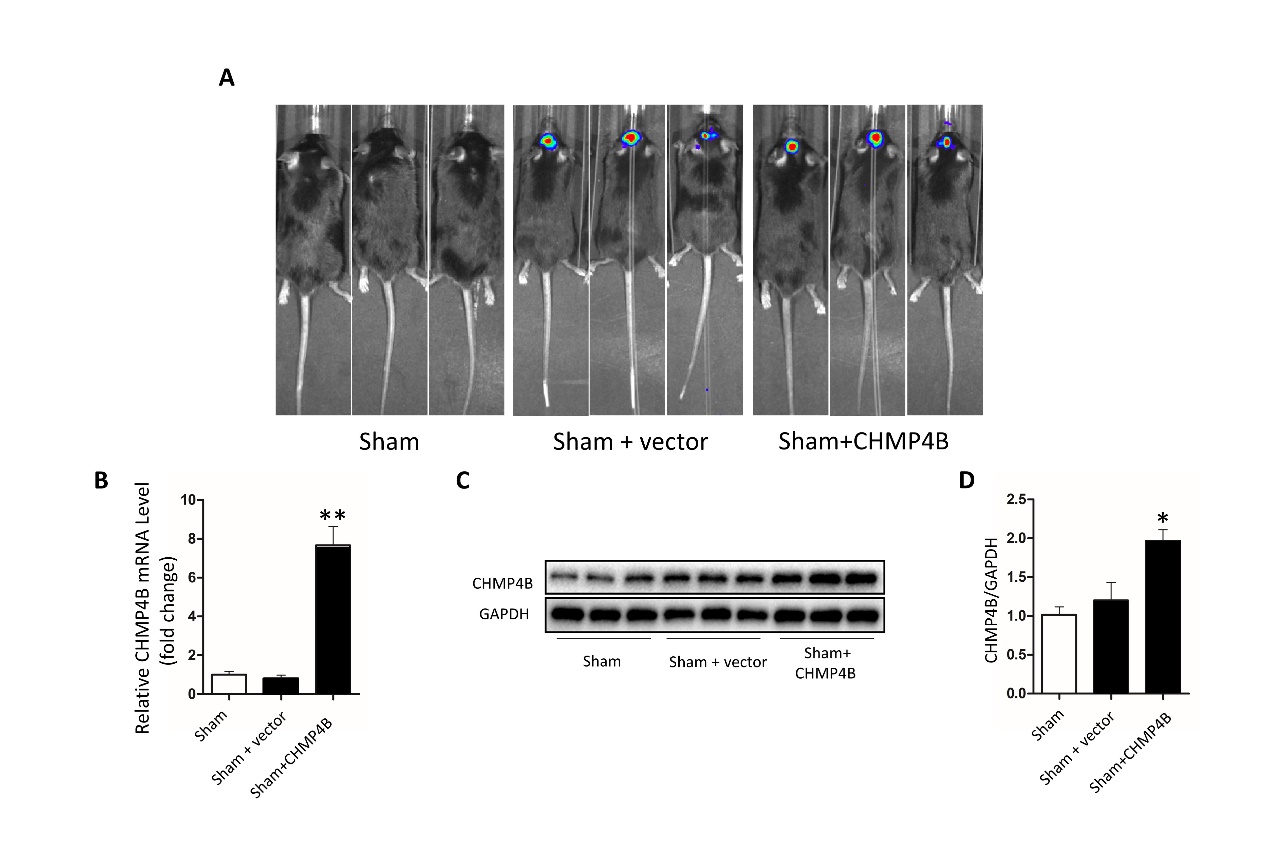


**SUPPLEMENTARY FIGURE 1**. AAV transfection of CHMP4B enhances or silences CHMP4B expression. mice were administered an intracerebroventricular injection of either AAV- luciferase or AAV-CHMP4B for a week before TBI. A. Optical in vivo imaging assay. B-D. The qRT-PCR and western blotting were conducted to test the transfection efficiency, and the data were statistically analyzed. (n=3 mice; data are presented as the means ± S.E.M.) *P < 0.05, **P < 0.01.


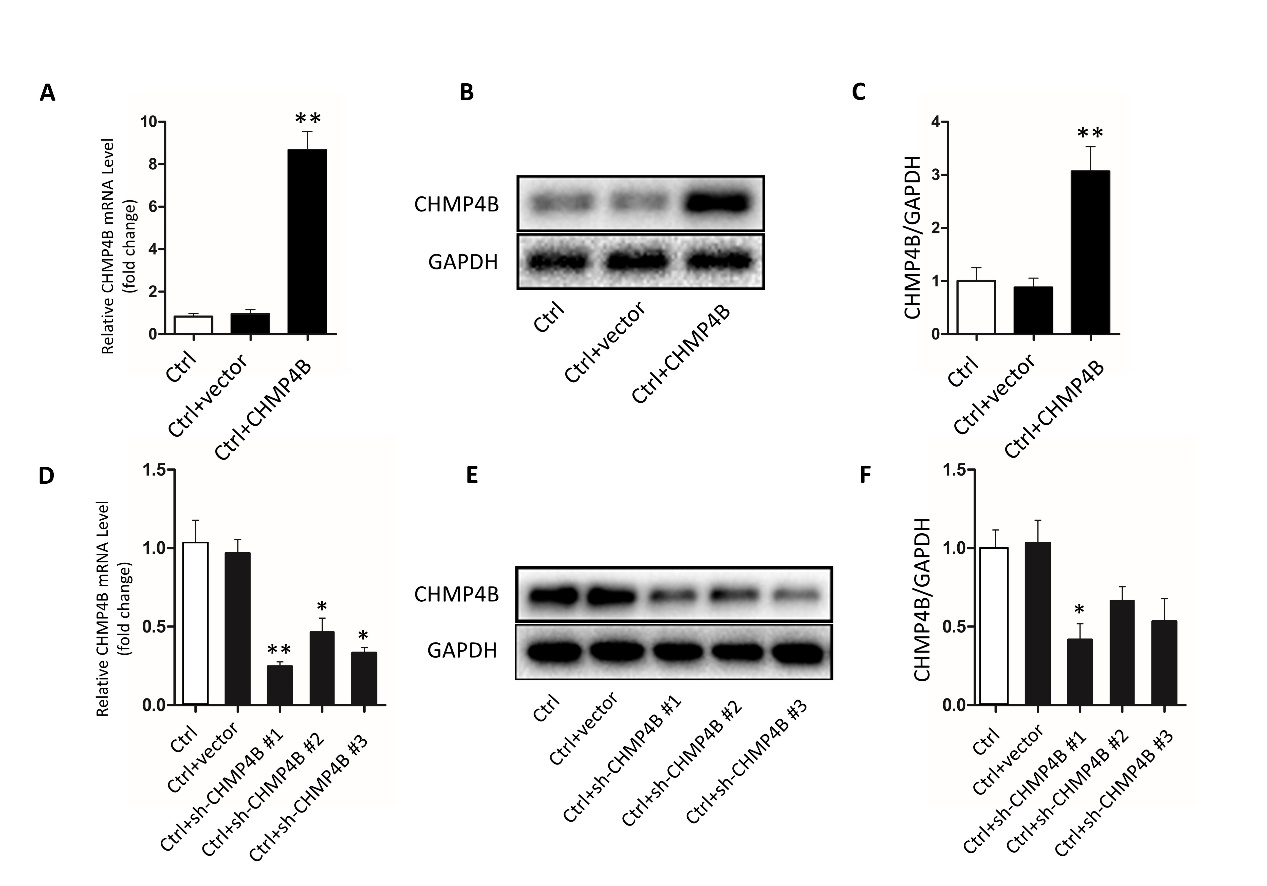


**SUPPLEMENTARY FIGURE 2**. We used plasmid to upregulate or knock down of CHMP4B in BV2 cells. The qRT-PCR and western blot assay was conducted to test the transfection efficiency, and the data were statistically analyzed (n=3; data are presented as the means ± S.E.M). *P < 0.05; **P < 0.01.


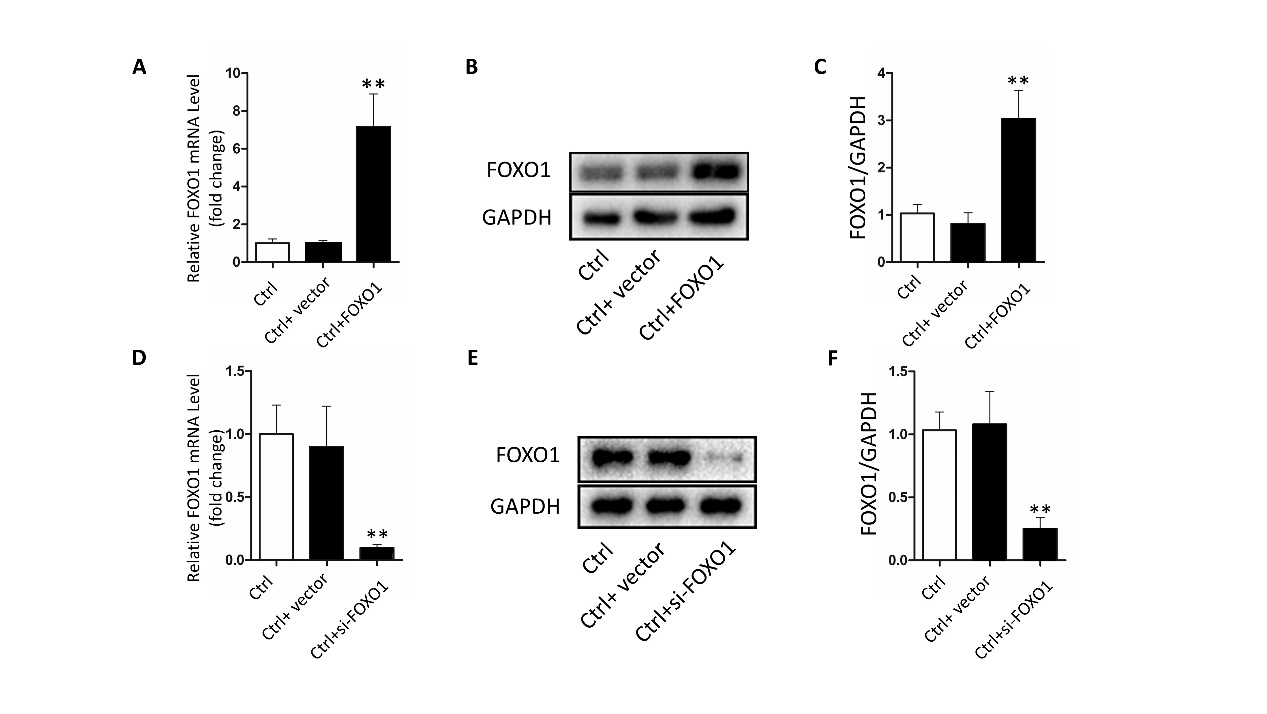


**SUPPLEMENTARY FIGURE 3**. We used plasmid to upregulate or knock down of FOXO1 in BV2 cells. The qRT-PCR and western blot assay was conducted to test the transfection efficiency, and the data were statistically analyzed (n=3; data are presented as the means ± S.E.M). **P < 0.01.


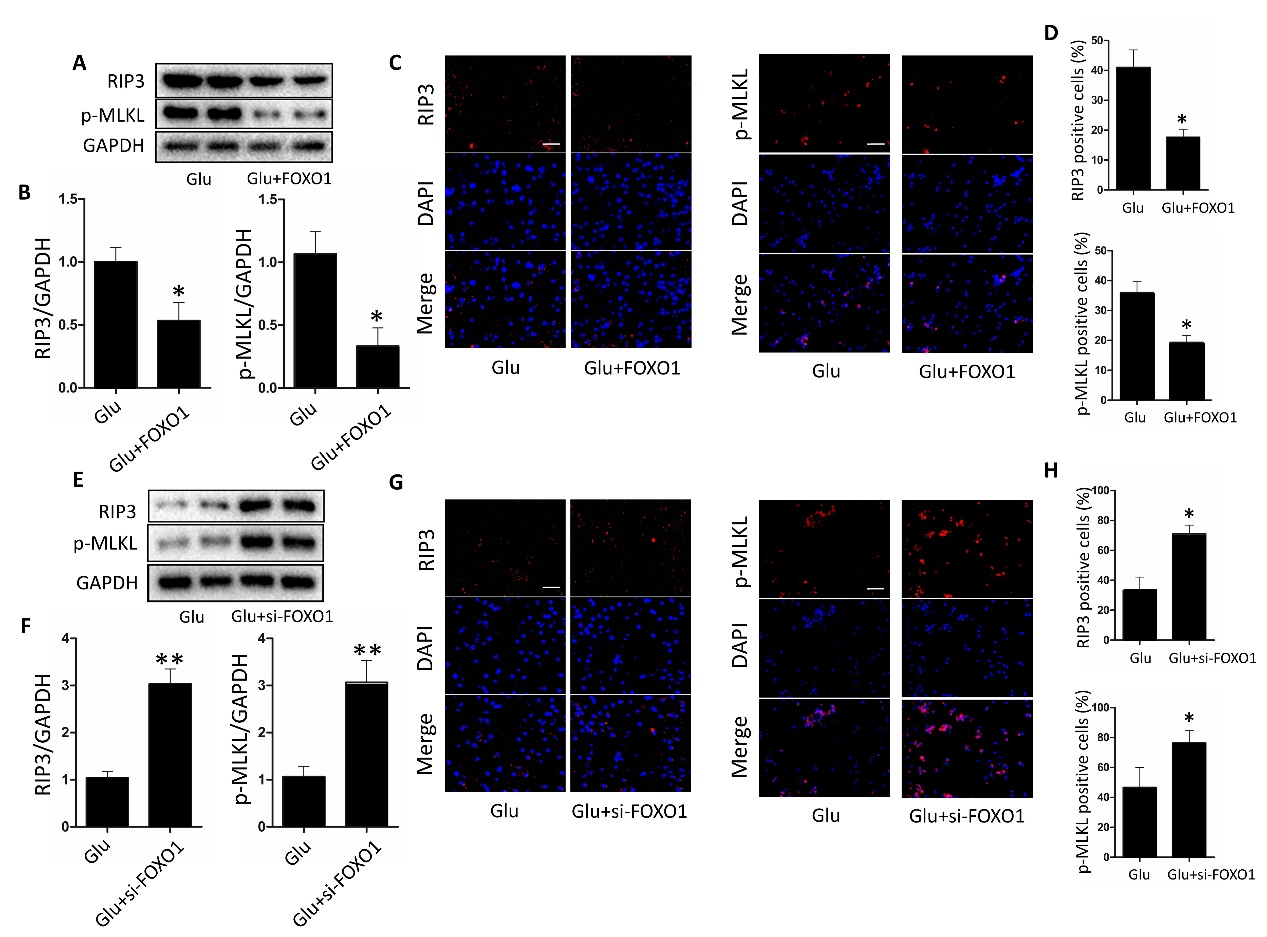


**SUPPLEMENTARY FIGURE 4.** In order to investigate the effect of FOXO1 on necroptosis, we transfected BV2 cells by plasmid to enhance or silence FOXO1 expression. A-D. western blotting assay and Immunofluorescence showed that FOXO1 overexpression inhibited necroptosis of BV2 cells after injury (n=3; Scale bar =100μm; data are presented as the means ± S.E.M). E-H. western blotting assay and Immunofluorescence showed that FOXO1 knockdown enhanced necroptosis of BV2 cells after injury (n=3; Scale bar =100μm; data are presented as the means ± S.E.M). *P < 0.05; **P < 0.01.
